# Cystic Neoplasms of the Exocrine Pancreas

**DOI:** 10.1155/1990/24762

**Published:** 1990

**Authors:** F. Mosimann, C. Ribaux, P. Schnyder

**Affiliations:** 1 Service de Chirurgie B Centre Hospitalier Universitaire Vaudois Centre Hospitalier Universitaire Vaudois Lausanne 1011 Switzerland; 2 lnstitut universitaire de Pathologie Centre Hospitalier Universitaire Vaudois Lausanne 1011 Switzerland; 3 Service de radiodiagnostic Centre Hospitalier Universitaire Vaudois Lausanne 1011 Switzerland

## Abstract

Cystic neoplasms of the pancreas are rare and their diagnosis and treatment can be difficult. This report details 7 patients who had histologically proven serous cystadenoma^4^, mucinous cystadenoma^2^ and cystadeno carcinoma^1^. Computed tomography and sonography allowed excellent preoperative assessment but to attempt a distinction between the histological variants may be hazardous. Two tumours were only autopsy findings and 5 patients underwent laparotomy. It is confirmed that potentially malignant mucinous cystadenomas and cytadenocarcinomas should be resected whenever possible; serous cystadenomas are always benign and should therefore be resected only when the diagnosis is doubtful or if they cause symptoms.


HPB Surgery, 1990, Vol. 2, pp. 165–175
Reprints available directly from the publisher
Photocopying permitted by license only

© 1990 Harwood Academic Publishers GmbH
Printed in the United Kingdom

### CYSTIC NEOPLASMS OF THE EXOCRINE PANCREAS

F. MOSIMANN[1*], C. RIBAUX[2], and P. SCHNYDER[3]
*Service de Chirurgie B, Centre Hospitalier Universitaire Vaudois, 1011 Lausanne[1] – Institut universitaire de Pathologie, Lausanne[2] – Service de radiodiagnostic[3], Centre Hospitalier Universitaire Vaudois, 1011 Lausanne, Switzerland*

*(Received 15th September, 1989)*

Cystic neoplasms of the pancreas are rare and their diagnosis and treatment can be difficult. This report details 7 patients who had histologically proven serous cystadenoma[4], mucinous cystadenoma[2] and cystadeno carcinoma[1]. Computed tomography and sonography allowed excellent preoperative assessment but to attempt a distinction between the histological variants may be hazardous. Two tumours were only autopsy findings and 5 patients underwent laparotomy. It is confirmed that potentially malignant mucinous cystadenomas and cytadenocarcinomas should be resected whenever possible; serous cystadenomas are always benign and should therefore be resected only when the diagnosis is doubtful or if they cause symptoms.

KEY WORDS: Pancreas, cystic tumours of the pancreas, serous cystadenoma, microcystic adenoma, glycogen-rich adenoma, mucinous cystadenoma, cystadeno-carcinoma

Cystic neoplasms of the exocrine pancreas are uncommon. They account for about 10% of all pancreatic cysts[1] and only 1% of pancreatic malignancies[2]. Due to their rarity the behaviour of these tumours is not well understood. It was only in 1978 that Compagno and Oertel were able to reduce the traditional classification into benign cystadenomas and malignant cystadenocarcinomas. After reviewing the files of the US Armed Force Institute of Pathology, they described two variants of cystic pancreatic tumours. The first one is the serous cystadenoma. It is benign and often referred to as microcystic adenoma or glycogen-rich adenoma[3]. The second variânt is the mucinous cystic neoplasm with latent or overt malignancy (mucinous cystadenoma and cystadenocarcinoma)[4]. Since these publications the new pathological classification has been confirmed in several smaller series and is now generally accepted[5,6,7,8,9,10,11,12]. Only a few hundred cases have however been described and several problems regarding clinical diagnosis and treatment remain therefore to be solved. This prompted us to analyse our experience with seven histologically proven serous or mucinous pancreatic cystadenomas.

### PATIENTS AND METHODS

The records of the departments of surgery and pathology of the Lausanne University Medical Centre from 1981 to 1987 were searched. Patients charts were

*Correspondence: F. Mosimann, Service de chirurgie B, Centre Hospitalier Universitaire Vaudois, 1011 Lausanne, Switzerland reviewed for clinical presentation, radiological evaluation, tumour localization, treatment, pathological examination and follow-up. All the operative and autopsy material was reviewed by a pathologist who was unaware of the radiologist's assessment and vice versa.

### RESULTS

Seven patients were identified. Four were females, 3 were males and the mean age was 69 years (range 37 to 82 years). None had a history of alcoholism, pancreatitis or abdominal trauma.

### *Clinical Presentation*

Two patients were asymptomatic and a serous cystadenoma was found incidentally at autopsy: a man had died of a cerebral vascular accident at 81 years of age and the other, an 82 year old woman, of complicated diverticular disease and a myeloproliferative syndrome. In a 37 year old female, the pancreatic mass was detected sonographically during the work up of acute epigastric pain: endoscopy revealed that this was due to an active duodenal ulcer rather than to the mucinous cystadenoma. In an 80 year old man, the diagnosis of pancreatic cystadenocarcinoma was also an incidental finding, as it was found during laparatomy for a rectal adenocarcinoma which proved to be non resectable and metastatic.

The remaining three patients, one man and two women aged 69, 82 and 53 years respectively had non specific complaints such as vague upper abdominal pain or discomfort. Two had gall-stones but their history did not suggest biliary pancreatitis attacks. Two noticed an abdominal mass by self-examination and one complained of a 6 kilos weight loss. Two of these symptomatic patients suffered a serous and one a mucinous cystadenoma. Two patients in this series had mild type II diabetes.

### *Radiological Evaluation*

CT scans and/or sonograms were available in four patients. They demonstrated in every case an encapsulated, lobulated, multicystic mass (Figure 1) with internal septations (Figure 2); a stellate central scar was seen on two occasions (Figure 3). Calcification was visible in two cases. The bile duct was never dilated and the pancreatic tissue surrounding the tumour was unremarkable. The adjacent organs were not infiltrated and the peripancreatic lymph nodes were not enlarged.

These findings were regarded as suggestive of a pancreatic cystic neoplasm but a distinction between a serous or mucinous cystadenoma was not attempted. At histology, two of these masses were serous and the other two mucinous tumours.

No patient underwent ERCP[13] or any other relevant radiological investigation.

### *Tumour Localization and Treatment*

Three tumours were in the head of the pancreas. A serous cystadenoma was an autopsy finding. The only cystadenocarcinoma in the series was simply biopsied because of a synchronous end-stage rectal adenocarcinoma. In the third patient a mucinous cystadenoma was drained into a Roux jejunal loop, because the surgeon

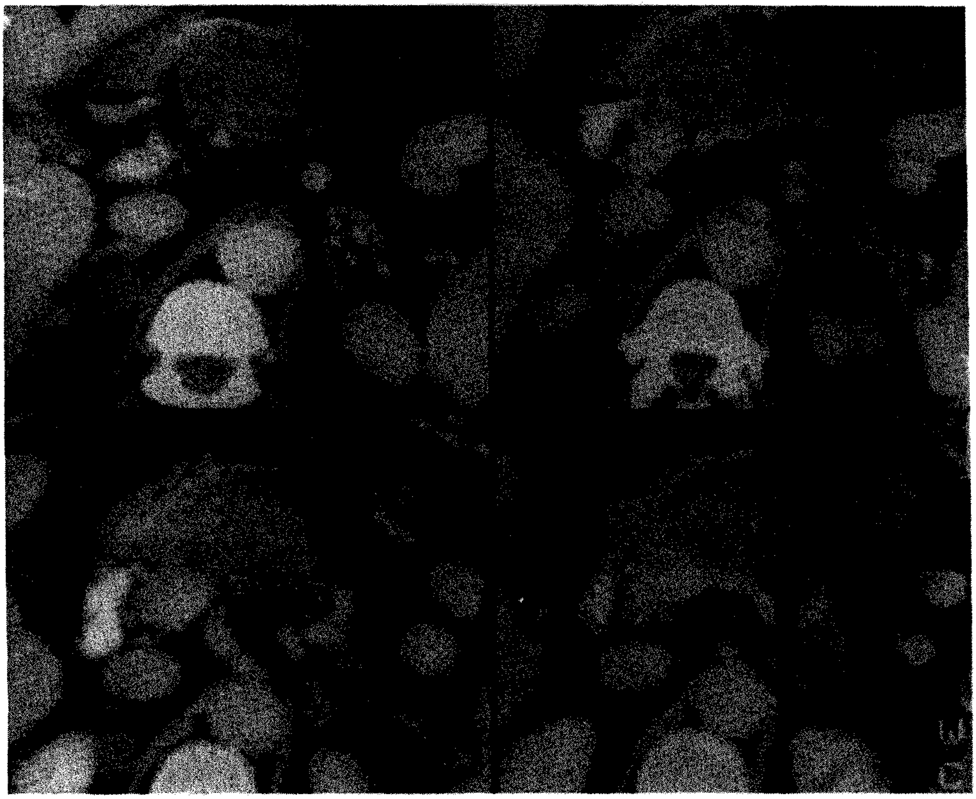

**Figure 1** CT. Encapsulated, lobulated multicystic mass in the pancreatic body (serous cystadenoma).

felt that a resection which would have included the superior mesenteric vessels was too hazardous, and because it was assumed that distension of the cyst by fluid might cause pain.

Of the four tumours located in the body (2) or tail (2), the largest was 15 cm in diameter. Three were resected by a left splenopancreatectomy (one mucinous and two serous) and a serous cystadenoma was found at autopsy.

*Pathology*

The serous cystadenomas were spherical tumours with an irregular surface due to the multilocular cysts. These varied in size from a few millimeters to about 2 cm in diameter and contained clear fluid. The cysts were separated from one another by fibrous tissue that sometimes formed a central stellate scar (Figure 4, same patient as Figure 3). Microscopically the cysts were lined by a cuboidal or flattened epithelium without well-formed papillae. The PAS-diastase stain showed intracytoplasmic glycogen (Figure 5). Mucins were not demonstrated in these tumours. The surrounding pancreatic parenchyma was normal.

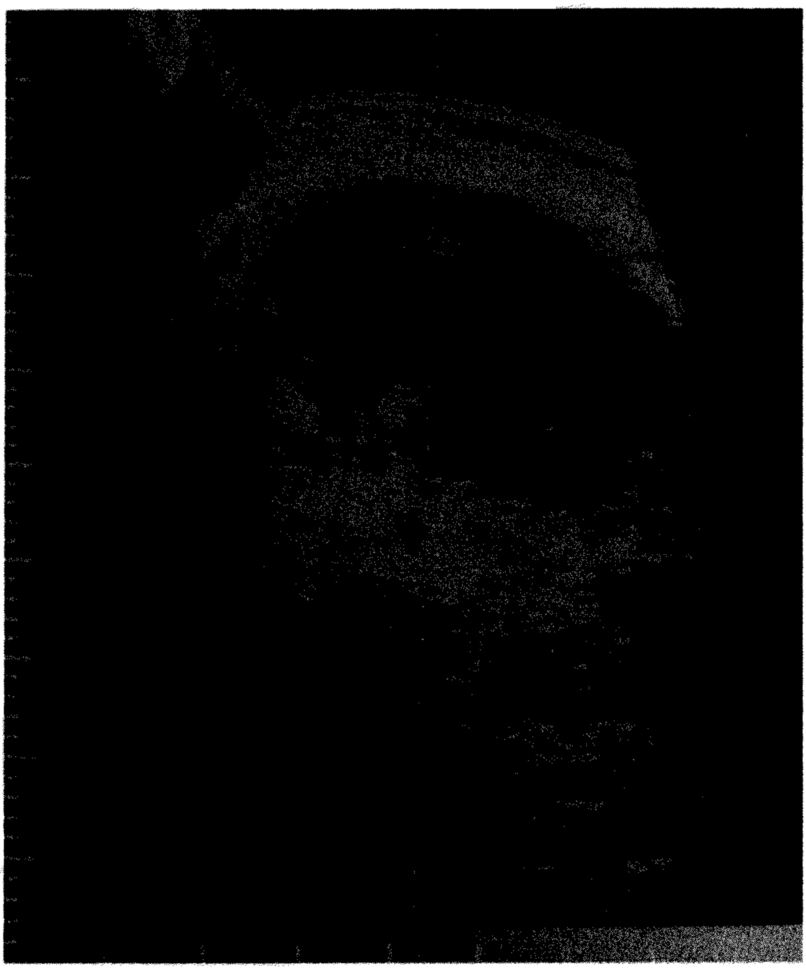

**Figure 2** Sonogram. Cystic mass in the pancreatic head, with internal septations (mucinous cystadenoma).

The mucinous cystadenomas were also spheric but their fibrous wall tended to be thicker than in the serous tumours. The cysts were also multiple but larger and less numerous than in the serous variant. They contained a whitish mucoid fluid. Microscopically their epithelium was columnar, sometimes forming papillae. Overt malignant changes were seen in only one case. Glycogen was not found but Alcian blue stain evidenced intracellular mucin (Figure 6).

*Follow up*

The patient with pancreatic cystadenocarcinoma and synchronous end-stage rectal adenocarcinoma died of cachexia and mechanical bowel obstruction, 4 ½ months after laparotomy. The other four operated patients had an uneventful postoperative course, although the two patients who had undergone splenopancreatic

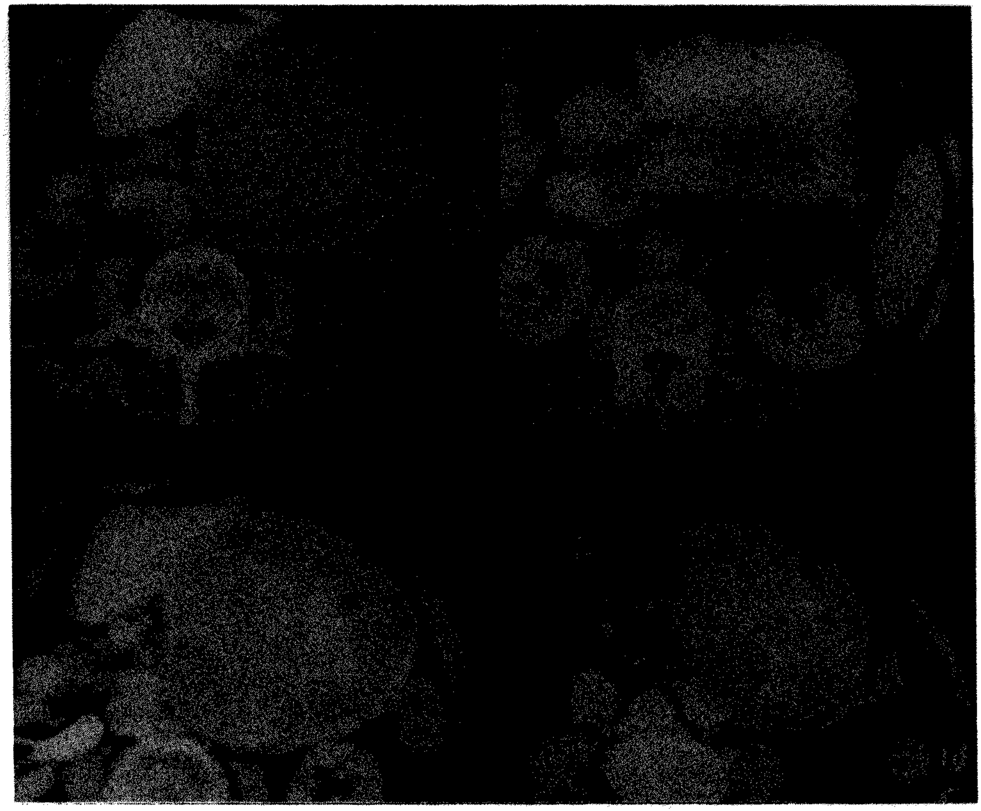

**Figure 3** CT. Encapsulated pancreatic cystic mass with a "stellate" central scar (serous cystadenoma).

resection for serous cystadenoma both developed a pseudocyst from the pancreatic section line within five months. Both were treated by cystogastrostomy without further complications. Histologic examination of the pseudocyst wall showed no epithelial lining in either case, thus ruling out recurrence of the cystadenoma. These two patients are asymptomatic three years after resection of the tumour. The youngest patient in the series, who had splenopancreatectomy for mucinous cystadenoma at 37 years, is also asymptomatic 5 years postoperatively. Finally the woman who had a mucinous cystadenoma drained into a Roux loop when aged 53 years, has no symptoms 5 years later. Her tumour has been stable in size at yearly CT scans.

### DISCUSSION

The most common cystic lesion of the pancreas is the pseudocyst. Unlike a tumour, it is generally associated with a history of pancreatitis or trauma. History however is of little help in reaching the diagnosis of cystic neoplasms because symptoms and

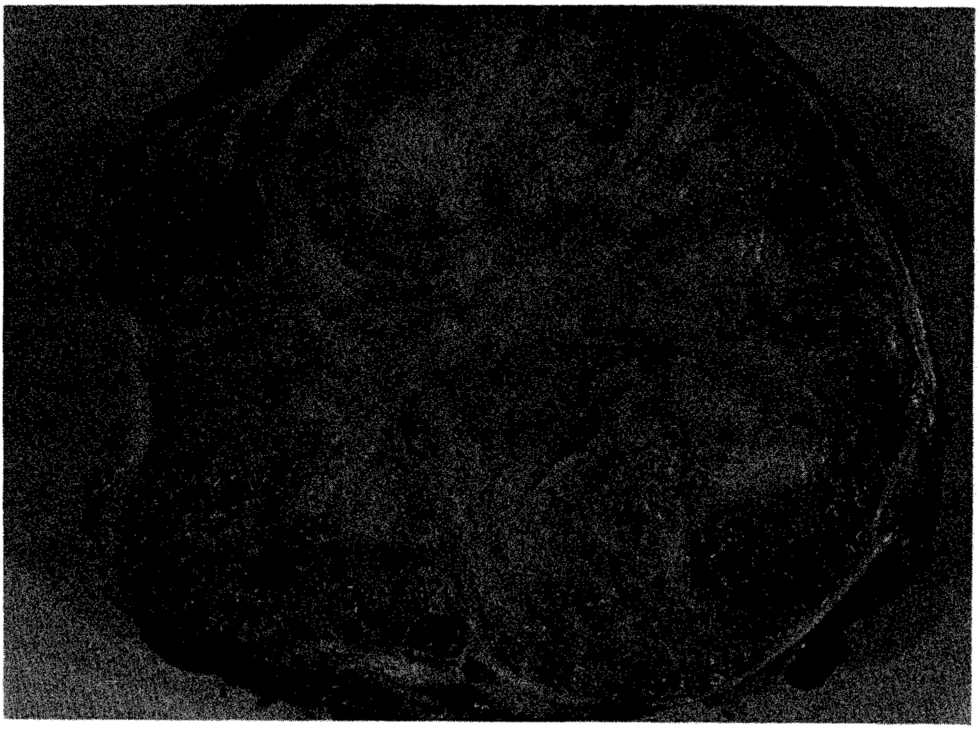

**Figure 4** Left splenopancreatic resection. The specimen shows a central "stellate" scar (Serous cystadenoma; same patient as Figure 3).

signs may be non-existent or minimal. When present they are always non-specific such as abdominal pain, epigastric discomfort or a palpable mass. Women may be slightly more susceptible than men[12,14] and most tumours occur late in life. Many conditions have been reported as sometimes being associated with cystadenomas, including hypertension, diabetes, thyroid dysfunction[15], gall-stones[16], von Hippel-Lindau's syndrome[17] and extrapancreatic neoplasms[3]. In the present series, two patients had both gall-stones and mild type II diabetes and two others had a rectal carcinoma and a myeloproleferative syndrome respectively. We feel however that these associations may be in fact coincidences in an elderly population.

In our experience laboratory tests have not been useful and the only radiological examinations that provided significant information were sonography and computed tomography. A typical serous cystadenoma is a spherical multilocular mass, often with a stellate central scar whereas a typical mucinous cystadenoma has a smoother surface, is uni or multilocular, has a denser fibrous wall and shows larger cysts. Although some radiologists[18,19] claim that these tumours present with a sufficiently characteristic pattern to allow the correct diagnosis to be made on the basis of CT, we consider that this diagnosis is presumptive. A recent report strengthens this view and alerts us to the fact that some pseudocysts, islet cell tumours, some ductal adenocarcinoma, papillary and solid epithelial neoplasms and pancreatic lymphangiomas may be indistinguishable from cystadenomas. Unlike Johnson *et al.*[20] we did

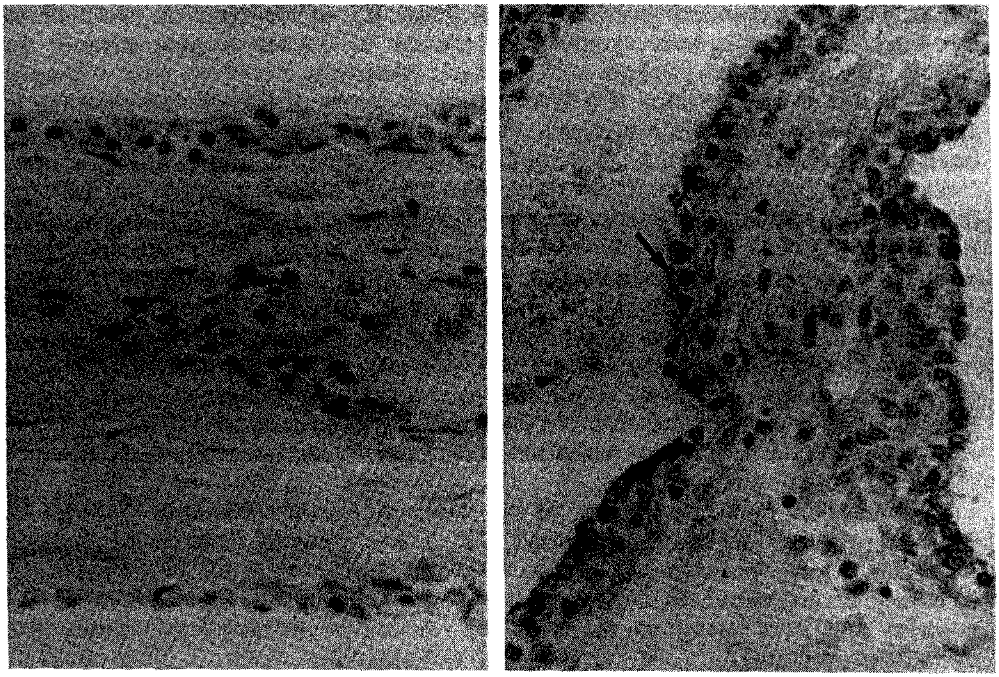

**Figure 5** Serous cystadenoma: histology. Left (HE): flattened epithelium. Right (PAS-diastase): intracytoplasmic glycogen is evidenced (arrow).

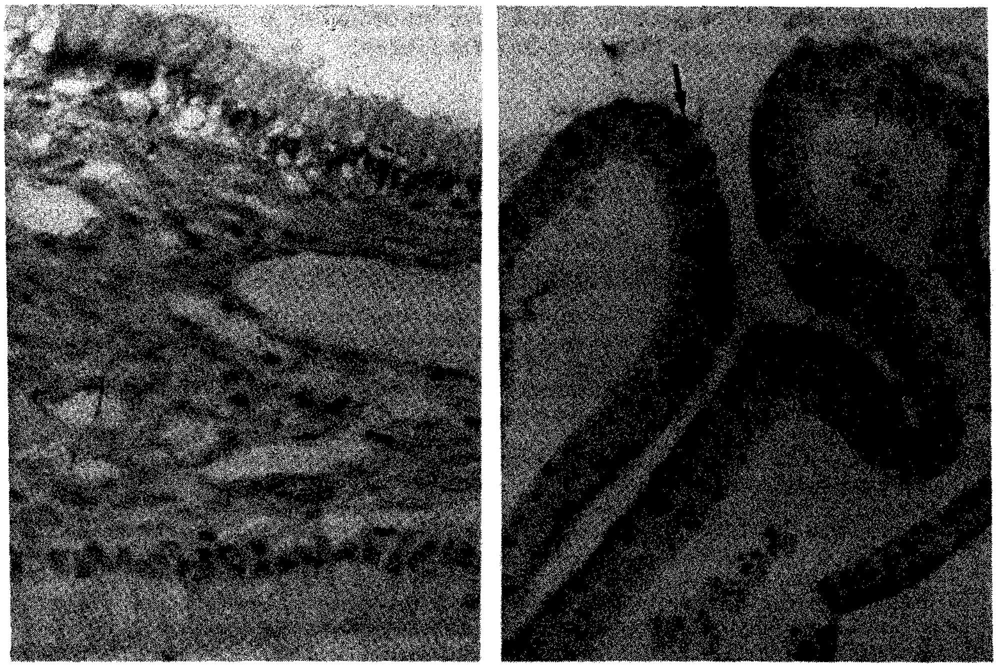

**Figure 6** Mucinous cystadenoma: histology. Left (HE): columnar epithelium. Right (Alcian blue): intracellular mucin is shown (arrow).

not perform percutaneous needle biopsies under CT or sonographic guidance as our operated patients were symptomatic enough to require surgery anyway. In elderly and debilitated patients with minimal symptoms however, such a biopsy may avoid a risky operation if a histological diagnosis of serous cystadenoma is established: such a tumour is always benign as no case of malignant change has ever been reported. In addition no intermediate variant between serous and mucinous cystadenoma has been described[14]. On the contrary should a biopsy lead to the diagnosis of mucinous cystadenoma or cystadenocarcinoma, laparotomy with pancreatic resection becomes the best option. Failure to resect the lesion may mean missing the opportunity to cure cancer, as benign and malignant epithelium may co-exist within the same tumour[4]. In some patients however the risk of a major procedure must be weighed against that of the slowly growing nature of these neoplasms[21,22,23]. This is why one of our patients, a 53 year old diabetic with gall-stones underwent a Roux loop drainage rather than a Whipple operation with resection of the superior mesenteric vessels: so far this less than ideal approach has resulted in an excellent 5 year palliation. There was no increase in tumour size at a recent CT check-up.

In conclusion, cystic neoplasms of the pancreas are rare tumours that can be detected at best by sonography and CT. These imaging techniques provide relatively typical pictures but an accurate diagnosis can only be reached by histology. If asymptomatic serous cystadenomas may be left alone, then on the contrary mucinous cystadenomas should be resected to prevent degeneration into cystadenocarcinoma.

### *Acknowledgements*

We acknowledge with grateful thanks the information provided by Prof. P. Hahnloser (Fribourg) and Prof. F. Gloor (St Gallen).

### *References*

1. Becker, W.F., Welsh, M.A. Prait H.S. (1965) Cystadenoma and cystadenocarcinoma of the pancreas. *Annals of Surgery* **161**, 845–863.
2. Cubilla, A.L., Fitzgerald, P.J. (1979) Classification of pancreatic cancer. *Mayo Clinic Proceedings*, **54**, 449–459.
3. Compagno, J., Oertel, J.E. (1978) Microcystic adenomas of the pancreas (Glycogen-rich cystadenomas). A clinicopathologic study of 34 cases. *American Journal of Clinical Pathology*, **69**, 289–298.
4. Compagno, J., Oertel, J.E. (1978) Mucinous cystic neoplasms of the pancreas with overt and latent malignancy (Cystadenocarcinoma and cystadenoma). A clinicopathologic study of 41 cases. *American Journal of Clinical Pathology*, **69**, 573–580.
5. Bogomoletz, W.V., Adnet, J.J., Widgren, S., Stavrou, M., McLaughlin, J.E. (1980) Cystadenoma of the pancreas: a histological, histochemical and ultrastructural study of seven cases. *Histopathology*, **4**, 309–320.
6. Segesser, L. von., Rohner, A. (1984) Pancreatic cystadenoma and cystadenomacarcinoma. *British Journal of Surgery*, **71**, 449–451.
7. Helpap, B., Wolff, P. (1985) Cystiche Pankreasneoplasien. *Chirurg*, **56**, 44–45.
8. Shorten, S.D., Hart, W.R., Petras, R.E. (1986) Microcystic adenomas (serous cystadenomas) of pancreas. A clinicopathologic investigation of eight cases with immunohistochemical and ultrastructural studies. *American Journal of Surgical Pathology*, **10**, 365–372.
9. Albores-Saavedra, Angeles-Angeles, A., Nadji, M., Henson, D.E., Alvarez, L. (1987) Mucinous cystadenocarcinoma of the pancreas. *American Journal of Surgical Pathology*, **11**, 11–20.
10. Kerlin, Frey, C.F., Bodai, B.I., Twomey, P.L., Ruebner, B. (1987) Cystic neoplasms of the pancreas. *Surgery, Gynecology and Obstetrics*, **165**, 475–478.

11. Warshaw, A.L., Rutledge, P.L. (1987) Cystic tumors mistaken for pancreatic pseudocysts. *Annals of Surgery*, **205**, 393–398.
12. Nordlinger, B., Etienne, J.C., Soua, N., Hervé, J.P., Hannoun, L., Frileux, P., Tiret, E., Huguet, C., Parc, R. (1988) Cystadénomes du pancréas. Lesquels réséquer ? 25 cas. *Chirurgie*, **114**, 641–647.
13. Delcenserie, R., Dupas, J.-L., Joly, J.-P., Descombes, P., Mortier, F., Capron, J.-P. (1988) Microcystic adenoma of the pancreas demonstrated by endoscopic retrograde pancreatography. *Gastrointestinal Endoscopy* **34**, 5254.
14. Yamaguchi, K., Enjoji, M. (1987) Cystic neoplasms of the pancreas. *Gastroenterology*, **92**, 1934–1943.
15. Soloway, H.B. (1965) Constitutional abnormalities associated with pancreatic cystadenomas. *Cancer*, **18**, 1297–1300.
16. ReMine, S.G., Frey, D., Rossi, R-L., Munson, L., Braasch, J.W. (1987) Cystic neoplasms of the pancreas. *Archives of Surgery*, **122**, 443–446.
17. Beerman, M.H., Fromkes, J.J., Carey, L.C., Thomas, F.B. (1982) Pancreatic cystadenoma in Von Hippel-Lindau disease: An unusual cause of pancreatic and common bile duct obstruction. *Journal of Clinical Gastroenterology*, **4**, 537–540.
18. Parienty, R.A., Ducellier, R., Lubrano, J.M., Picard, J.D., Pradel, J., Smolarski, N. (1980) Cystadenomas of the pancreas: Diagnosis by computed tomography. *Journal of Computed Tomography*, **4**, 364–367.
19. Wolfman, N.T., Ramquist, N.A., Karstaedt, N., Hopkins, M.B. (1982) Cystic neoplasm of the pancreas: CT and sonography. *American Journal of Roentgenology*. **138**, 37–41.
20. Johnson, C.D., Stephens, D.H., Charboneau, J.W., Carpenter, H.A., Welch, T.J. (1988) Cystic pancreatic tumors: CT and sonographic assessment. *American Journal of Roentgenology*, **151**, 1133–1138.
21. Hodgkinson, D.J., ReMine, W.H., Weiland, L.H. (1978) Pancreatic cystadenoma. A clinicopathologic study of 45 cases. *Archives of Surgery*, **113**, 512–519.
22. Taft, D.A., Freeny, P.C. (1981) Cystic neoplasms of the pancreas. *American Journal of Surgery*, **142**, 30–35.
23. Hyde, G.L., Davis Jr. J.B., McMillin, R.D., McMillin, M. (1984) Mucinous cystic neoplasm of the pancreas with latent malignancy. *American Surgeon*, **50**, 225–229.

### INVITED COMMENTARY

Most cysts of the pancreas are "false", because they have no epithelial lining. These pseudocysts arise from inflammation or trauma causing obstruction, distension and rupture of the pancreatic ductal tree. The 10–20 per cent of "true" cysts are usually neoplastic, occasionally congenital and exceptionally parasitic or endometriotic; however, their epithelial lining is often shed, at least in part. Among cystic tumours there are two types of cystadenoma (carcinoma), as described in the present report, plus a fascinating condition called papillary cystic neoplasm (solid and papillary neoplasm) and rarities such as lymphangioma and acinar cell cystadenocarinoma. In addition exocrine (ductal) adenocarcinoma of the pancreas can occasionally be cystic and so can endocrine (islet cell) tumours, whether benign or malignant. Zamora's historical review of pancreatic cystic tumours makes interesting reading. In early reports they were likened to ranulas ("genouillettes" or little frogs), the retention cysts that develop in the floor of the mouth from obstruction of a salivary duct. Indeed, the pancreas was regarded as the salivary gland of the abdomen during the early 1800s.

Dr Mosimann and his colleagues stress several important points about cystic neoplasms of the pancreas:

1. They have a female predilection. Serous cystadenoma affects elderly women, mucinous cystadenoma/carcinoma affects middle-aged women and papillary cystic neoplasm is virtually confined to young women. This distribution is unexplained, though the recent findings of progesterone receptors in a couple of papillary cystic neoplasms suggests a possible sex hormone dependence[2].

2. The pathological sub-types of cystadenoma are of great clinical importance. Mucinous cystadenoma has a clearcut malignant potential; one of three cases in the present series showed evidence of cystadenocarcinoma. By contrast, serous cystadenoma or microcystic adenoma does not appear to undergo malignant change. Thus if a confident diagnosis of serous cystadenoma could be made without laparotomy, and if the patient were aged and frail and also free of symptoms, a good case could be made for leaving the tumour alone[3]. Unfortunately, preoperative diagnosis is fallible (see below), and percutaneous biopsy in this context is largely untried. Other things being equal, therefore, a resectable pancreatic tumour should still be resected.

3. These are indolent tumours that often present as incidental findings (as with 4 of the 7 Swiss cases). Ultrasound and especially CT scanning is invaluable; the scans reveal a circumscribed cystic tumour within the pancreas. Enthusiasts claim to be able to distinguish the serous and mucinous patterns not only from one another but from the other types of cystic tumour[4,5]. Though readily accepting that some of these tumours may have pathognomonic appearances, I remain sceptical about the sensitivity of radiological discrimination. Little weight can be attached to features such as calcification, hypervascularity or even metastasis, since these can accompany most of the mimicking tumours[2]. More promising may be the ability to differentiate benign and malignant cysts according to the absence or presence of tumour markers in cyst fluid obtained by percutaneous needle aspirations[6].

4. There is another trap for the unwary. A cystic neoplasm may be mistaken for a pseudocyst and treated inappropriately by internal drainage or (worse still) by percutaneous drainage. Warshaw describes eight such cases[7]. In one of the present patients with mucinous cystadenoma, internal drainage was intentionally performed because of the proximity of the superior mesenteric vessels. The authors state that resection would have entailed sacrificing these vessels, but I am surprised. If the tumour were truly benign, a plane of dissection should readily have been demonstrable. If malignant, then internal drainage was surely inappropriate, despite the apparently successful outcome in their particular patient. I have personally resected one cystic neoplasm (an epithelioid leiomyoma) in a patient whose "pseudocyst" obstinately "recurred" after drainage at another hospital first into the stomach and subsequently into a Roux loop of jejunum[8]!

### *References*

1. Zamora, J.L. (1985). Cystic neoplasms of the pancreas. Evolution of a concept. *American Journal of Surgery* **149**, 819–823.
2. Williamson, R.C.N. (1989). Endocrine and other unusual pancreatic tumours. Current opinion in *Gastroenterology* **5**, in press.
3. Algard, M., Ponsot, P., Hautefeuille, M., Menu, Y., Fekete, F., Paolaggi, J-A. (1986). *Gastroenterologie Clinique et Biologique* **10**, 23–28.
4. Choi BI, Kim, K.W., Han, M.C., Kim, YI., Kim, C-W. (1988). Solid and papillary epithelial neoplasms of the pancreas: CT findings. *Radiology* **166**, 413–416.
5. Itai, Y., Ohhashi, K., Furui, S., Araki, T., Murakani, Y., Ohtomo, K., Atomi, Y. (1988). Microcystic adenoma of the pancreas: spectrum of computed tomographic findings. *Journal of Computer Assisted Tomography* **12**, 797–803.

6. Tatsuta, M., Iishi, H., Ichii, M., Noguchi, S., Yamamoto, R., Yamamura, H., Okuda , S. (1986). Values of carcinoembryonic antigen, elastase 1, and carbohydrate antigen determinant in aspirated pancreatic cyst fluid in the diagnosis of cysts of the pancreas. *Cancer* **57**, 1836–1839.
7. Warshaw, A.L., Rutledge, P.L. (1987). Cystic tumors mistaken for pancreatic pseudocysts. *Annals of Surgery* **205**, 393–398.
8. Gillatt, D.A., Cooper, M.J., Williamson, R.C.N. (1986). Pseudo-pseudocysts of the pancreas. *Pancreas* **1**, 460–463.

R.C.N. Williamson
Hammersmith Hospital
London, UK

